# Melting dsDNA Donor Molecules Greatly Improves Precision Genome Editing in *Caenorhabditis elegans*

**DOI:** 10.1534/genetics.120.303564

**Published:** 2020-09-22

**Authors:** Krishna S. Ghanta, Craig C. Mello

**Affiliations:** * RNA Therapeutics Institute, University of Massachusetts Medical School, Worcester, Massachusetts 01605; † Program in Molecular Medicine, University of Massachusetts Medical School, Worcester, Massachusetts 01605; ‡ Howard Hughes Medical Institute, University of Massachusetts Medical School, Worcester, Massachusetts 01605

**Keywords:** CRISPR, HDR, Genome Editing, Donor DNA

## Abstract

CRISPR genome editing has revolutionized genetics in many organisms. In the nematode *Caenorhabditis elegans*, one injection into each of the two gonad arms of an adult hermaphrodite exposes hundreds of meiotic germ cells to editing mixtures, permitting the recovery of multiple indels or small precision edits from each successfully injected animal. Unfortunately, particularly for long insertions, editing efficiencies can vary widely, necessitating multiple injections, and often requiring coselection strategies. Here, we show that melting double-stranded DNA (dsDNA) donor molecules prior to injection increases the frequency of precise homology-directed repair (HDR) by several fold for longer edits. We describe troubleshooting strategies that enable consistently high editing efficiencies resulting, for example, in up to 100 independent GFP knock-ins from a single injected animal. These efficiencies make *C. elegans* by far the easiest metazoan to genome edit, removing barriers to the use and adoption of this facile system as a model for understanding animal biology.

## Abstract (precis)

Melting and fast cooling double stranded DNA donor molecules prior to injection dramatically increases the frequency of homology-directed repair for edits such as insertions of fluorescent protein markers in *Caenorhabditis elegans*. Strategies described here enable consistently .....

IN the nematode worm *Caenorhabditis elegans*, genome editing can be achieved by direct injection of Cas9 guide-RNA ribonucleoprotein (RNP) complexes into the syncytial ovary (Cho *et al.* 2013; Paix *et al.* 2015; Dokshin *et al.* 2018). In the worm germline, such injections afford the editing machinery simultaneous access to hundreds of meiotic germ nuclei that share a common cytoplasm. Under optimal conditions, the frequency of F1 progeny with indels caused by nonhomologous end joining (NHEJ) can be >90% of those progeny expressing a co-injection plasmid marker gene (Dokshin *et al.* 2018). Leveraging these high cutting efficiencies, precise genome editing is readily achieved using short [under ∼200 nucleotide (nt)], single-stranded oligodeoxynucleotide (ssODN) donors, permitting insertions of up to ∼150 nt in length (Arribere *et al.* 2014; Zhao *et al.* 2014; Paix *et al.* 2015; Prior *et al.* 2017; Dokshin *et al.* 2018). However, with longer dsDNA donors (∼1 kb), homology-directed repair (HDR) events are recovered at lower frequencies, require more complex protocols, high concentrations of the donor DNA, and typically require screening the broods of multiple injected animals (Tzur *et al.* 2013; Arribere *et al.* 2014; Kim *et al.* 2014; Ward 2015; Paix *et al.* 2016, 2017; Schwartz and Jorgensen 2016; Dokshin *et al.* 2018; Farboud *et al.* 2019; Silva-García *et al.* 2019; Vicencio *et al.* 2019).

There are multiple reasons why longer repair templates may be less efficient as donors for HDR compared to ssODNs. First, empirical studies suggest that long dsDNA is more toxic than short single-stranded DNA (Mello *et al.* 1991), limiting safe donor concentrations to less than 200 ng/µl for ∼1 kb donors. Second, upon injection into germline cytoplasm, dsDNA molecules quickly form large extra-chromosomal arrays via both end-joining and homologous recombination pathways, and appear to do so while sequestered away from genomic DNA (Stinchcomb *et al.* 1985; Mello *et al.* 1991). Concatenation of donor molecules into large arrays would have the effect of lowering the number of individual molecules available to access and to template repair at the target site double-strand break (DSB). Moreover, if injected DNA assembles concatenates while sequestered from the nuclear DNA—perhaps within *de novo* nucleus-like organelles (Forbes *et al.* 1983)—this process could preclude templated repair of a genomic target site until after the sequestered concatenates gain nuclear access after nuclear envelope breakdown occurs postfertilization.

In a recent study, we showed that CRISPR-mediated HDR could be increased ∼fourfold by mixing, melting, and reannealing overlapping donor molecules to create donor templates with single-stranded overhangs (Dokshin *et al.* 2018). In those previous studies, we limited our analysis to a cohort of F1 “Roller” progeny that express the co-injection marker gene *rol-6** (**su1006**)*. Here, to explore editing efficiency outside the Roller cohort, we scored the entire brood of each injected animal for precisely edited progeny that incorporate and express fluorescent protein markers (GFP or mCherry). We show that the vast majority of insertions occurred later in the brood, after the cohort of progeny that express the Roller phenotype. Whereas overhangs improved the frequency of editing among the F1 Rollers (Dokshin *et al.* 2018), they had no benefit within this latter segment of the brood. Instead, melting the donor molecules, alone, sufficed to increase the HDR frequency to as high as 50% of the postinjection progeny. We provide a protocol and troubleshooting strategies that enable even a novice injector to achieve their editing goals and to optimize editing efficiencies.

## Materials and Methods

The detailed editing protocol is provided in Supplemental Material, File S1.

### Strains and genetics

All the strains were generated in the Bristol N2 background unless specified otherwise and cultured on normal growth media (NGM) plates seeded with OP50 bacteria (Brenner 1974). Strains used in this study are listed in Table S1.

At the CSR-1 locus, GFP was introduced between FLAG::linker (9 bp) and TEV in FLAG::linker::TEV::CSR-1 strain.

### Scoring methodology

Injected P0 animals were cultured individually on NGM plates at room temperature (22–23°) unless specified otherwise. P0 animals with more than 100 postinjection progeny and at least 20 Rollers were selected—except at 100 ng/µl and 200 ng/µl of dsDNA donor where number of Rollers can be lower than 20 due to toxicity—and their F1 progeny were scored between 72 and 90 hr postinjection. All the F1 progeny from each brood were mounted onto 2% agarose pads and screened under fluorescence microscope for GFP or mCherry expression. GraphPad Prism (version 8.4) was used to perform statistical tests and calculate P-values.

### Oligos and donors

End-modified donors were generated by PCR using oligos with 5′ SP9 modifications (IDT). Oligos used for to generate *hrde-1* and *F53H1.1** gfp* donors also contain 15 bp linkers on either end of *gfp*, which also serve as PCR primers. Sequences of all the crRNAs and oligos are provided in Table S2 and Table S3, respectively.

### Data availability

The authors state that all data necessary for confirming the conclusions presented in the manuscript are represented fully within the manuscript. All the reagents are available upon request. Supplemental material available at figshare: https://doi.org/10.25386/genetics.12984149.

## Results

### Melting the donor dramatically stimulates HDR for longer edits

We recently showed that melting and reannealing donor molecules to create asymmetric donors with single-stranded homology arms can improve the frequency of CRISPR-mediated homology-directed insertions among transformants that were positive for a transformation marker (Dokshin *et al.* 2018). Because transformation markers can cause confounding effects or toxicity, we decided to conduct an initial study in which markers were omitted altogether. For this purpose, we chose to target the insertion of *gfp* into the easily scored *glh-1* locus, which encodes a VASA-related DEAD-box protein that localizes robustly to germline perinuclear foci known as P granules or nuage.

**Figure 1:**
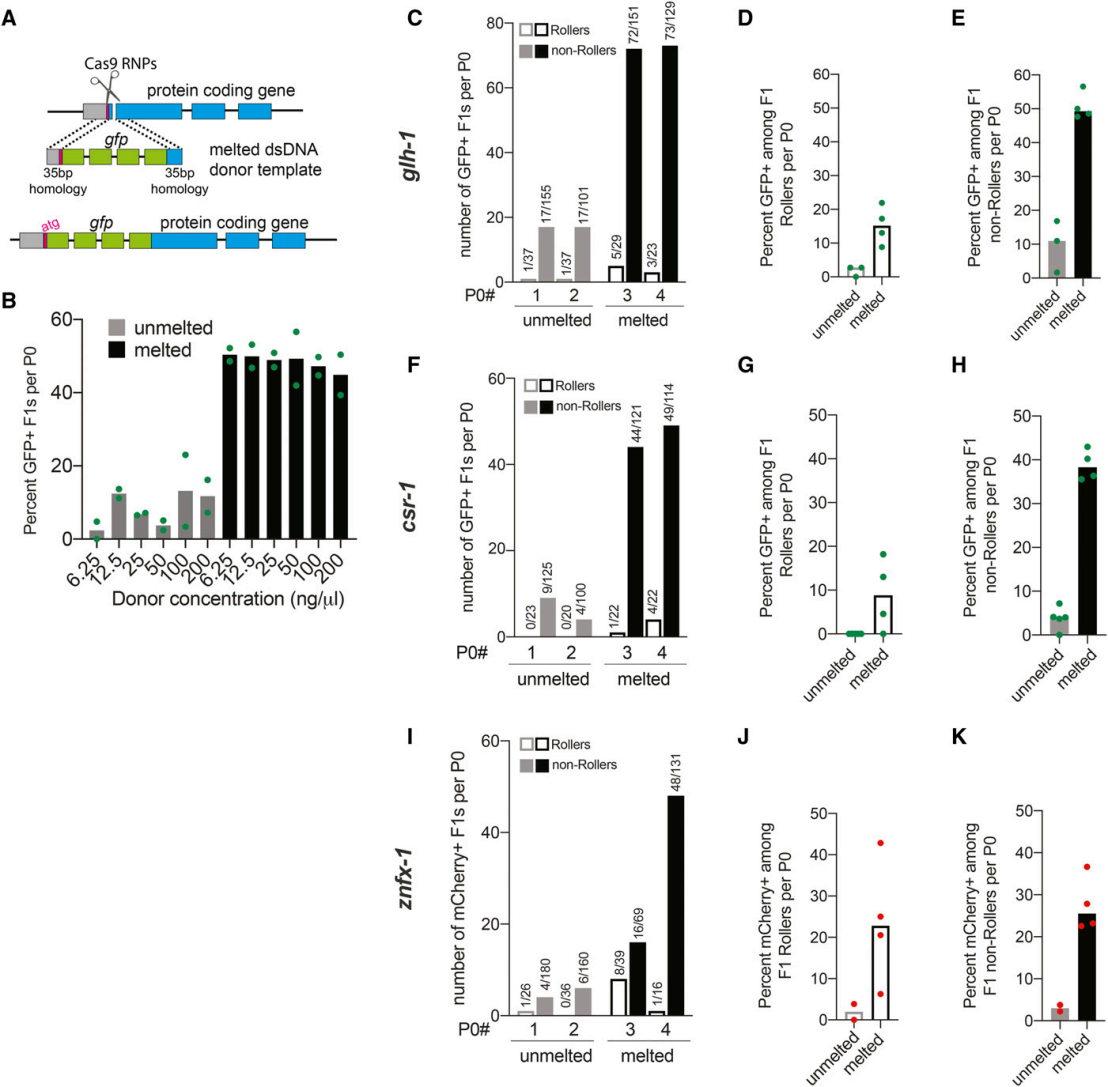
Melting dsDNA donors potentiates homology-directed repair (HDR) in *C. elegans*. (A) Schematic representation to insert *gfp* at the N-terminus of a protein-coding gene immediately downstream of start codon (ATG) using symmetric melted dsDNA donors and Cas9-guideRNA ribonucleoproteins (RNPs) is shown; gray segment represents sequence upstream of the start codon. Precise repair (HDR) enables fluorescent protein expression. (B) HDR efficiencies at the *glh-1* locus using symmetric unmelted (gray bars) or melted donors (black bars) with *rol-6* injection marker at indicated concentrations (*n* = 2 broods) is plotted as percentage of F1s expressing GFP per injected animal (P0). Using unmelted and melted donors, HDR efficiencies at the *glh-1* locus is plotted as (C) number of fluorescence+ animals among Rollers and non-Rollers from two representative broods. Percentage of animals expressing fluorescence among, (D) Rollers and (E) non-Rollers, is plotted as percentage (*n* = three or four broods) for *glh-1* locus. Similarly, improvement in fluorescent protein insertion efficiencies with melted donors are shown for (F–H) *csr-1* and (I–K) *znfx-1* loci. Each data point represents the percentage of animals expressing fluorescent protein among F1s scored in each cohort per brood. Bars represent median. Number of fluorescence+ animals over number of animals scored is shown above the bars. Green dots represent GFP and red dots represent mCherry insertions.

We prepared the *gfp* donor by PCR using primers tailed with 35 nt of homology to the *glh-1* locus (Figure 1A). In order to separately analyze the consequences of melting and of generating single-stranded overhangs we prepared three types of donor, (i) PCR products that were never melted, “unmelted donors,” (ii) “melted donors” that were heated and allowed to reanneal, and (iii) “asymmetric melted donors” that were prepared by heating a mixture of two overlapping *gfp* PCR products [one with 35-bp homology to *glh-1* at each terminus and one without (Dokshin *et al.* 2018)]. For simplicity, we refer to denaturing and quickly cooling the donor as “melting,” (see *Materials and Methods*). We injected each type of donor along with Cas9-guide-RNPs targeting *glh-1* into the core cytoplasm of the pachytene syncytium just distal to the gonad turn. Ideal injections result when the flow of the injection solution extends bilaterally from the injection site into the queue of oocytes at the proximal end and into the mitotic region at the distal end (Mello *et al.* 1991). Only animals with two such injections—one per arm—were analyzed.

As previously shown (Dokshin *et al.* 2018), the asymmetric melted donor outperformed the unmelted donor. The asymmetric *gfp*::*glh-1* donor yielded 381 GFP-positive transformants among 900 F1 progeny, or 42% of total postinjection progeny. The unmelted symmetric donor in contrast yielded half as many edits, 161 GFP-positive transformants among 740 postinjection progeny, (22%). Surprisingly, the symmetric melted donor was just as effective as the asymmetric melted donor, yielding 331 GFP positives among 906 F1 progeny, (37%). Thus, when the entire brood is scored melted symmetric donor was as effective as its asymmetric counterpart. For melted donors, the number of GFP positive edits equaled approximately two-fifths of all postinjection progeny exceeding the total number of Roller transgenics typically recovered per injected animal (Figure S1 and see below).

### Efficient HDR occurs over a broad range of donor concentrations

To explore how the frequency of *gfp* edits varied over a range of donor concentration, we injected unmelted or melted *gfp*::*glh-1* donor at concentrations of 6.25, 12.5, 25, 50, 100, and 200 ng/µl (25 ng = 0.04 pmol). In order to control for injection quality, each injection mix included 40 ng/µl of the *rol-6**(**su1006**)* cotransformation marker. For each donor mix, we injected five to seven worms, singled those receiving optimal bilateral injections, and further analyzed two worms that made at least 100 postinjection progeny, including at least 20 Rollers. We then screened all the postinjection progeny—Roller and non-Roller—for germline GFP expression. We noted that the overall percentage of *gfp* insertions per injected animal (40–50% for melted donors) (Figure 1B) was similar to levels achieved when the *rol-6* marker was omitted (Figure S1), suggesting that the *rol-6* marker does not interfere with the overall efficiency of editing. Surprisingly, the frequency of GFP-positive progeny per injected animal remained similar over a 32-fold range of donor concentrations. Melted donors consistently outperformed unmelted donors at every concentration (Figure 1B). These results suggest that, even at the donor concentration of 6.25 ng/µl, the HDR efficiency may be near saturation. At donor concentrations above 25 ng/µl, the frequency of Rollers per injected animal declined, suggesting that these higher concentrations cause toxicity (Figure S2). Taken together, these findings suggest that melted donors provide high rates of HDR with low toxicity over donor concentrations in the range of 6.25 ng/µl (0.01 pmol/µl) to 25 ng/µl (0.04 pmol/µl). Based on these findings we chose to use 25 ng/µl of donor in further investigations.

We next wished to examine how editing efficiencies vary among the Roller and non-Roller cohorts of postinjection progeny. We found that melted donors outperformed unmelted donors in both Roller and non-Roller cohorts (Figure 1, C–E), yielding several dozen *gfp* edited progeny per injected animal (as shown in two representative broods, Figure 1C). Strikingly, the fraction of GFP expressing progeny was much higher among non-Rollers (49%) (Figure 1E) compared to Rollers (15%) (Figure 1D).

To confirm the generality of these findings, we targeted two additional germline-expressed genes: *csr-1* and *znfx-1* (Figure 1, F-K). In both cases, melted donors consistently outperformed unmelted donors for *gfp* and *mCherry* insertions respectively (Figure 1, F and I**)**. When melted donors were used, the fraction of animals with precision insertions was ∼10-fold higher than levels obtained with unmelted donors. This enhancement was observed in both the Roller (Figure 1, G and J) and non-Roller cohorts (Figure 1, H and K**)**. We also explored whether melted donors were beneficial for editing with Cas12a (CPF1) (Ebbing *et al.* 2017) RNPs — which recognize an AT rich TTTV protospacer adjacent motif (PAM) sequence. Indeed, Cas12a editing yielded high HDR efficiencies comparable to those achieved with Cas9 RNPs for *gfp* insertion at both the *glh-1* and *F53H1.1* loci (Figure S3) (See File S1 for protocol).

### Editing efficiency peaks later in the brood after the roller cohort of progeny are produced

**Figure 2:**
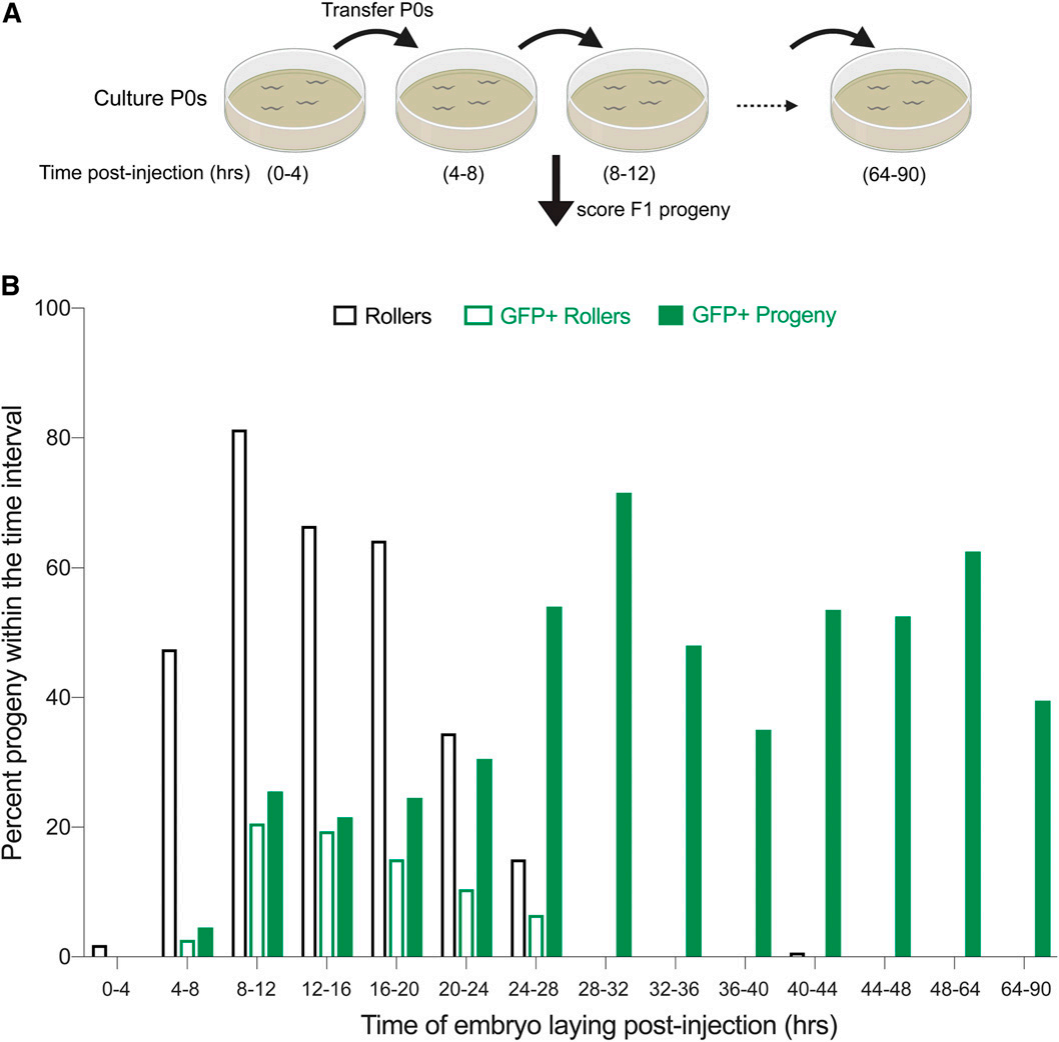
Editing occurs later in the brood after roller cohort. (A) Schematic representation of the experiment is shown. Four injected animals placed on a single plate were moved at indicated postinjection time points and F1 embryos laid during the time-intervals were scored for GFP as adults. (B) Fraction of the progeny produced in each time window that are Rollers (open black bars), GFP+ Rollers (open green bars) and GFP+ progeny (Rollers and non-Rollers, solid green bars) are plotted as percentage. Bars represent mean value of two replicates and each replicate consists of four P0 animals injected with 25 ng/µl of symmetric melted donors and 40 ng/µl of *rol-6* co-injection marker. Animals were cultured at 18–20°.

The finding that HDR events are more prevalent among non-Roller progeny might reflect different developmental competencies of germ nuclei to form these distinct types of transgenics. For example, distal pachytene germ nuclei may be more receptive to recombination between the target chromosomal locus and the *gfp* donor, whereas more proximal germ nuclei may be more receptive to forming extrachromosomal transgenes driven by recombination between co-injected DNA molecules (see *Discussion*) (Mello *et al.* 1991). To examine these possibilities, we followed the production of Roller and GFP-positive progeny over the entire postinjection brood. Worms receiving ‘ideal’ bilateral injections of an editing mix prepared with melted *gfp*::*glh-1* donor (25 ng/µl) and *rol-6* co-injection marker (40 ng/µl) were cultured in two groups of four injected animals. Each group of animals was transferred every 4 hr to fresh plates to divide their broods into 12 segments over the next 2 days. Animals were transferred one more time on the third day (64 hr postinjection) thus dividing the progeny into 14 groups (Figure 2A). We then scored the frequency of Roller progeny and GFP-positive progeny in each segment.

Consistent with the idea that Roller extrachromosomal transgenes assemble in more proximal germ cells, nearly 100% of the Roller progeny were produced within the first 28 hr post injection. The frequency of Rollers peaked between 8 and 12 hr postinjection where Rollers comprised 81% of the 47 progeny produced in the interval. The frequency of Roller progeny remained ∼60% until 20 hr post injection, declining to ∼30% then 13% over the next two 4-hr intervals. Rollers were virtually absent among progeny produced after 28 hr (Figure 2B). In striking contrast, the frequency of precision editing events was low within the first 24 hr, and then appeared to plateau and remain high during the entire remainder of the brood (Figure 2B). For example, only 20% of the 306 progeny produced in the first 24 hr were GFP positive while an average of 54% were positive among the progeny produced thereafter (*n* = 1327). Importantly, while GFP precision editing was less frequent within the first 24 hr (where Roller transgenics were found), precision editing was not under-represented within the Roller cohort. For example, we found that 24% of Rollers *vs.* 20% of all animals produced in the first 24 hr were GFP positive (Figure 2B). Moreover, among GFP positive animals produced in this interval 60% were Rollers. Thus, the Roller marker positively correlates with *gfp* editing but does so within a cohort of progeny that precedes the optimal editing window for *gfp* insertion (see *Discussion*).

### Donor purity is crucial for best HDR efficiencies

**Figure 3:**
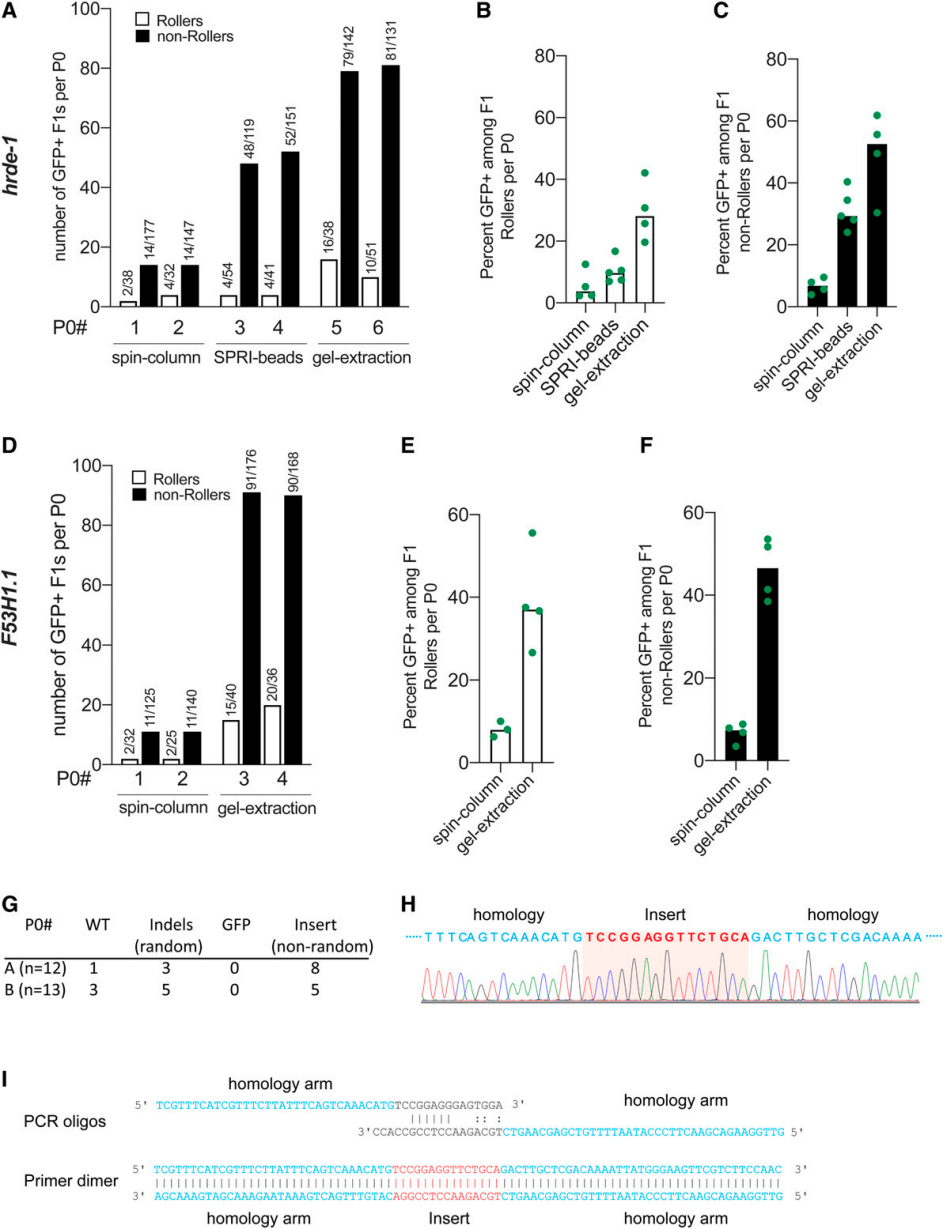
Purity of donor DNA is crucial for best HDR efficacy. HDR efficiencies of donors prepared by different methods of purification are plotted for *hrde-1* and *F53H1.1*. (A) Number of GFP+ F1 Rollers and non-Rollers from two representative broods are plotted. GFP+ animals among, (B) Rollers, and (C) non-Rollers is plotted as percentage of animals scored in each cohort per brood. Similarly, (D–F) HDR efficiencies are plotted for *F53H1.1* locus. (G) Insertions and deletions identified at *hrde-1* target site in F1 Rollers from two P0s (spin-column), (H) Sanger sequencing trace of the 15 bp nonrandom insert for a homozygous F2 animal. Partial homology arms of the donor are shown in blue, and the sequence that got inserted into the genome is shown in red. (I) Schematic representation of predicted primer dimer formation is shown with 6 bp perfect match and mismatched 3′ tails. Part of each oligo that is homologous to the PCR template plasmid is shown in black (linkers on either end of *gfp*) and the homology arms are shown in blue and the sequence (Insert) that would get inserted through HDR is shown in red. All the donors were 5′ TEG-modified and melted. Gel-purified donors were further cleaned-up with SPRI beads (see File S1).

Although *rol-6* transformation precedes the optimal window of *gfp* insertion (as shown above), we nevertheless found that the *rol-6* marker provides a valuable troubleshooting metric (Figure S2). For example, while attempting to knock-in *gfp* at two different loci (*hrde-1* and *F53H1.1*), gfp insertions were unexpectedly rare. These experiments were conducted using melted TEG-modified donors (Ghanta *et al.* 2018), which typically yield as many as 100 GFP+ progeny per injected worm. However, despite ideal injections that produced high numbers of Roller progeny, only two (average) Rollers were GFP positive per brood (spin-column, Figure 3, A and D). Scoring entire broods for GFP, we obtained a maximum of only 18 (*hrde-1*) (Figure 3A, P0# 2) and 13 (*F53H1.1*) (Figure 3D, P0#s 1 and 2) GFP-positive progeny per injected worm. The fraction of Rollers (spin-column, Figure 3, B and E**)** or non-Rollers (spin-column, Figure 3, C and F**)** expressing GFP stayed < 8% at both the loci. Because the number of Rollers per injected animal was near the optimal range, we reasoned that the injection quality was good, injected animals were healthy, and the injection mixture was nontoxic.

To understand why editing was so infrequent, we sequenced the target site in 25 randomly selected F1 rollers. In 21 of 25 Rollers, we identified nonwild-type sequences at the target site (Figure 3G), indicating that DSBs were not the limitation. Importantly, none of these 21 Rollers contained *gfp* insertions (Figure 3G). Upon reading the sequencing trace, we found that 13 F1 animals contained a 15-bp insertion precisely where *gfp* sequences should have inserted (Figure 3G and 3H). To our surprise, this short sequence perfectly matched a segment of the PCR oligo sequences (Figure 3I), and, thus, could be explained by insertion of a primer fragment or primer-dimer that was produced inadvertently during donor preparation. To test this possibility, we purified the *gfp* donors by size-exclusion using solid phase reversible immobilisation (SPRI) paramagnetic beads or by gel-extraction. Purifying the *hrde-1* donor with SPRI paramagnetic beads (optimized to exclude fragments < 300 bp) modestly increased the percentage of GFP-positive progeny to 10% of F1 Rollers (*n* = 212; Figure 3B) and 32% of non-Roller progeny (*n* = 625; Figure 3C). By contrast, gel-purified *hrde-1* donor dramatically increased the percentage of GFP-positive progeny to 29% of F1 Rollers (*n* = 163; Figure 3B) and 49% of non-Rollers (*n* = 538; Figure 3C), with as many as 95 GFP-positive progeny from one injected worm (Figure 3A, P0#5). Similar results were obtained after gel purification of the *F53H1.1* donor (Figure 3, D–F). These findings demonstrate the utility of the Roller marker as a metric for troubleshooting the editing protocol and reveal the importance of removing PCR-based contaminants from donor preparations to achieve best knock-in efficiencies.

## Discussion

We initiated these investigations to explore why long (∼1 kb) DNA donors were less efficacious than short ssODN donors in *C. elegans*. We have shown that melting the donor DNA dramatically enhances precision editing, enabling efficient editing with shorter homology arms and at significantly lower donor DNA concentrations than previously recommended (Paix *et al.* 2015, 2017; Dokshin *et al.* 2018). We show that as many as 100 precisely edited progeny can be obtained from a single injected animal—an editing efficiency of nearly 50% of postinjection progeny, and far exceeding the typical frequency of progeny transformed with simple extrachromosomal arrays (Figure S2) (Mello *et al.* 1991).

Importantly, whereas the production of Roller transgenic progeny peaks during the first 24 hr postinjection, *gfp* edits peak after 24 hr and remain high through the remainder of the injected animals brood. Previous studies also reported that most *gfp*-edited animals are produced on the second day after injection (Paix *et al.* 2014). These findings suggest that developmental differences between distal (less mature) and proximal (more mature) germ nuclei may favor formation or acquisition of distinct transgene types. For example, perhaps the large *rol-6* plasmid molecules are excluded from germ nuclei, and instead rapidly assemble into cytoplasmic extrachromosomal arrays that are swept by the germ plasm into developing oocytes, and only enter nuclei after fertilization [as previously suggested (Mello *et al.* 1991)]. A size limitation on nuclear uptake may explain why we and others have found that donors over 2 kb yield few editing events (unpublished results) (Paix *et al.* 2016; Farboud *et al.* 2019).

The observation that *gfp* editing peaks later, approximately 28 hr postinjection, and then remains high, suggests either that proximal germ nuclei tend to exclude the donor, or that the pachytene nuclei are more receptive to recombination. Each gonad arm of a young adult worm contains hundreds of meiotic nuclei at the time of injection and each injected animal produces <200 postinjection progeny; therefore, it is likely that all the postinjection progeny come from the pachytene nuclei. Furthermore, based on an ovulation rate of 23 min (McCarter *et al.* 1999), the appearance and persistence of GFP-positive progeny is consistent with editing in nuclei that were in pachytene (*i.e.*, undergoing meiotic recombination) rather than in the mitotic zone at the time of injection. Whatever the reason for the HDR enhancement caused by melting the donor, it is striking that the extrapolated rates of precision *gfp* insertion within these pachytene nuclei range as high as 70%.

Donor purity is crucial to achieve high knock-in efficiencies of long inserts. Contaminating primer dimers that contain homology arms can compromise HDR efficiency by integrating at the target site. Removing these contaminants by gel-extracting the donors dramatically increased *gfp* knock-in efficiencies. Similarly, as a time saving alternative to gel-extraction, we found that purification using SPRI paramagnetic beads also improves HDR efficiencies; however, using the optimal ratio of beads to PCR reaction was critical to removing the shorter contaminants (see protocol File S1).

We do not know why melting the donor stimulates HDR. We obtained similar HDR rates across the entire range of donor concentrations, indicating that donor concentrations were saturated (or nearly so) at the lowest dose tested. Yet, melting the donor increased the HDR rate several fold at each concentration. Thus, melting stimulates recombination by acting on events or mechanisms that are independent of donor concentration. Conceivably, melting induces structural changes—*e.g.*, denaturation bubbles caused by incomplete reannealing—that promote active nuclear uptake or directly stimulate repair. For example, single-stranded regions from incomplete reannealing could promote strand invasion or act as damage signals that recruit *trans*-acting factors that facilitate HDR. Indeed, consistent with the idea that incomplete reannealing may be important preliminary studies utilizing slow cooling to promote better reannealing, resulted in about half as many *gfp* insertions as fast-cooled donors (Figure S4). However, further studies of this issue are required as the *P*-value in this initial study was not statistically significant. Interestingly, melting did not stimulate the already high HDR efficiency of a shorter 400-nt donor (data not shown). Clearly more work is needed to fully explore and understand how donor-melting promotes HDR efficiency.

Undoubtedly, the high efficiencies of precision editing achieved here owe both to the easy access of worm pachytene germ cells to microinjection, and to the remarkable receptiveness of these cells to HDR. A parallel study suggests that editing is enhanced even further when donor 5′ ends are modified with tri-ethylene glycol (TEG) (Ghanta *et al.* 2018). Importantly, the combination of melting and TEG modifications increases the proportion of *gfp*-sized edits among the easily identified Roller progeny cohort by ∼20-fold from 1%–2% to 20%–40%. For experienced injectors, a single optimally injected animal can yield more than 100 GFP knock-ins (nearly two-thirds of postinjection progeny), dramatically enhancing the ease and efficiency of genome editing. Given these high HDR efficiencies, even researchers with little worm experience can now readily adopt this facile genetic animal model.
